# The effect of vitamin C deficiency and chronic ultraviolet-B exposure on corneal ultrastructure: a preliminary investigation

**Published:** 2011-11-26

**Authors:** Sally Hayes, Thamara A. Cafaro, Patrycja J. Boguslawska, Christina S. Kamma-Lorger, Craig Boote, Jonathan Harris, Robert Young, Jennifer Hiller, Nicholas Terrill, Keith M. Meek, Horacio M. Serra

**Affiliations:** 1 School of Optometry and Vision Sciences, Cardiff University, Cardiff, United Kingdom; 2 CIBICI, Faculty of Chemistry, Universidad Nacional de Córdoba, Argentina; 3 Diamond Light Source, Didcot, Oxford, United Kingdom

## Abstract

**Purpose:**

In the visually debilitating condition of climatic droplet keratopathy, corneal transparency is progressively lost. Although the precise cause of the disease and the mechanism by which it progresses are not known, a lifetime exposure to high solar radiation and a vitamin C–deficient diet may be involved in its development. This study examines the effect of dietary ascorbate levels and ultraviolet (UV)-B exposure on corneal stromal structure.

**Methods:**

Eight guinea pigs were divided into four treatment groups (A, B, C, and D). For 15 weeks, Groups A and C were fed an ascorbate-rich diet (2 mg/100 g bodyweight/day), while Groups B and D received an ascorbate-deficient diet (0.07 mg/100 g bodyweight/day). For the last 12 weeks of the study, Groups C and D also experienced chronic UVB exposure (0.12 J/cm^2^ for 40 min/day). Following euthanasia, the corneas were enucleated and their stromal ultrastructure examined using X-ray scattering and electron microscopy.

**Results:**

UVB exposure resulted in an increased corneal thickness (p<0.001), but this was not accompanied by a widespread expansion of the collagen fibrillar array, and in the case of ascorbate-deficient animals, stromal thickening was associated with the compaction of collagen fibrils (p<0.01). Neither UVB exposure nor ascorbic acid deficiency caused any change in the average diameter or D-periodicity of the stromal collagen fibrils.

**Conclusions:**

UVB-induced changes in the corneal ultrastructure were most pronounced in animals fed an ascorbic acid–deficient diet. This suggests that ascorbic acid may play a vital role in protecting the corneal stroma from the harmful effects of UVB.

## Introduction

Chronic exposure to ultraviolet (UV)-B light (290–320 nm) has been implicated in the development of several ocular disorders, such as pterygium [1,2], cataracts [1], and climatic droplet keratopathy [2,3]. Laboratory-based studies have shown that wavelengths below 290 nm (UVC) are almost completely absorbed by the corneal epithelium, whereas UVB penetrates deeper into the tissue and is absorbed by the corneal stroma and lens [4,5]. Corneal damage induced by chronic UVB exposure is thought to occur through a series of photooxidation reactions that result in the production of free radicals and reactive oxygen species. The presence of ascorbic acid in the corneal epithelium [6] is believed to limit damage to deeper ocular structures by absorbing UV radiation [7]. This is supported by the naturally high concentration of ascorbic acid in the corneas of diurnal species (especially those exposed to high solar radiation) compared to nocturnal species [8], as well as the reduced severity of corneal damage in UVB-exposed rabbits that are pretreated with ascorbic acid [9]. Moreover, in Norway, it was reported that corneal haze following photorefractive keratectomy (in which the ascorbate-rich epithelial layer is removed) only occurred during the summer months when the sun was visible 24 h per day [10], but when pre- and postoperative supplementation of vitamin C was used, the incidence of corneal haze reduced from 3.7% to zero [11]. We have also found that climatic droplet keratopathy patients in Patagonia (Argentina) experience a lifetime of constant intense winds, high solar radiation, and low humidity [12], and have abnormally low levels of ascorbate in their blood as a result of a low vitamin C diet (unpublished data).

In addition to its role as an antioxidant, ascorbic acid is also a cofactor in collagen synthesis reactions, where it is specifically needed for the hydroxylation of proline and lysine [13]. Without the hydroxylation of these amino acids, procollagen is unable to crosslink properly to form stable collagen fibrils [14], and glycosidic linkages between collagen and specific glycosaminoglycans may not be established [15]. Disturbing the precise organization of collagen in the corneal stroma may result in a loss of tissue transparency, since this property is largely dependent on the presence of uniformly narrow fibrils lying parallel to each other in layers (lamellae), which are themselves organized in a lattice-like configuration [16-18].

To determine the effect of chronic UVB exposure and ascorbate deficiency on the corneal stromal ultrastructure, electron microscopy images were obtained from specific sites in the tissue, and small-angle X-ray scattering was used to produce average, full-tissue thickness measurements of collagen fibril separation distance, fibril diameter, and D-periodicity (the axial distance by which each collagen molecule within a fibril is staggered with respect to its neighbors) [19]. Guinea pigs were used in this study because, like humans, they are diurnal and lack the enzyme L-gulonolactone oxidase; ascorbic acid must therefore be obtained from the diet to fulfill its role as an antioxidant and as a cofactor in collagen synthesis reactions.

## Methods

### Animals

This study was performed in accordance with the Association for Research in Vision and Ophthalmology Statement on the Use of Animals in Ophthalmic and Vision Research.

Eight healthy male albino guinea pigs (Statens Serum Institut, Allerød, Denmark) aged between 6 and 7 months were used for the purposes of this study. For 15 weeks, the animals were housed in pairs, in stainless steel cages with wood shavings on the floor. All animals were exposed to illumination from ceiling lights (36 W daylight fluorescent tubes) on a 12 h:12 h light-dark cycle, and the housing environment was maintained at a temperature of 23 °C and a humidity of 60% (National University of Cordoba, Argentina). Water and homemade guinea pig pellets containing alfalfa, soybeans, oats, and wheat were available to all animals ad libitum, but the level of vitamin C intake was regulated by means of a single measured daily dose via a micropipette. Animals in Group A (n=2) and C (n=2) received the normal recommended ascorbic acid dose for guinea pigs (2 mg/100 g bodyweight/day), while the animals in Groups B (n=2) and D (n=2) received a low dose of ascorbic acid (0.07 mg/100 g bodyweight/day). Following a three week pretreatment period, which served to deplete the body stores of ascorbic acid in Groups B and D, animals in Groups C and D were exposed to a daily 40 min dose of UVB (0.12 J/cm^2^/day) for the remainder of the study (12 weeks).

The treatment groups and their abbreviations are summarized below:

Group A: Normal ascorbate without UVB exposure (AA+, UVB-)
Group B: Ascorbate deficient without UVB exposure (AA-, UVB-)
Group C: Normal ascorbate with 40 min daily UVB exposure (AA+, UVB+)
Group D: Ascorbate deficient with 40 min daily UVB exposure (AA-, UVB+)

Prior to treatment and twice a month during the study, the corneas of each animal were examined using a slit-lamp biomicroscope (Led Slit Lamp XL-1; Shin Nipon, Ohira Co., Niigata, Japan) with 10× magnification to evaluate the presence/absence of epithelial erosions. Biomicroscopy was performed before and after the topical instillation of 2.5% sodium fluorescein (Laboratories Poen, Buenos Aires, Argentina). Fluorescein staining of the cornea was measured in arbitrary units (0=no colorant uptake, 1=discrete uptake, 2=moderate uptake, 3=marked uptake).

Measurements of corneal thickness were recorded at weeks 1 (pretreatment period), 5, 10, and 15 using an ultrasonic pachymeter SP100 (Tomey Corp., Nagoya, Japan). In each case, the measurements were taken 2 h before the daily UVB exposure of Groups C and D. Bodyweight was measured at weekly intervals.

Following treatment, the guinea pigs were euthanized with an overdose of ketamine [20], after which the corneas with a 2–3 mm scleral rim were removed from each eye and a small suture placed in the sclera at the 12 o’clock position. The corneas were then wrapped tightly in thin plastic film (Rolopac^TM^; BANDEX, Buenos Aires, Argentina) to prevent tissue dehydration, labeled, and frozen at −80 °C. The frozen corneas were then transported to Cardiff University (UK) on dry ice.

### X-ray data collection

Immediately before data collection, each cornea (wrapped in Rolopac^TM^) was defrosted at room temperature. The cornea was then rewrapped in a single layer of Clingfilm^TM^ (Superdrug Stores, Croydon, UK) and mounted in its correct orientation (scleral suture at the 12 o’clock position) in a sealed polymethyl methacrylate chamber (Perspex; theplasticshop.co.uk, Coventry, UK) with polyester film windows (Mylar; DuPont-Teijin, Middlesbrough, UK).

**Figure 1:**
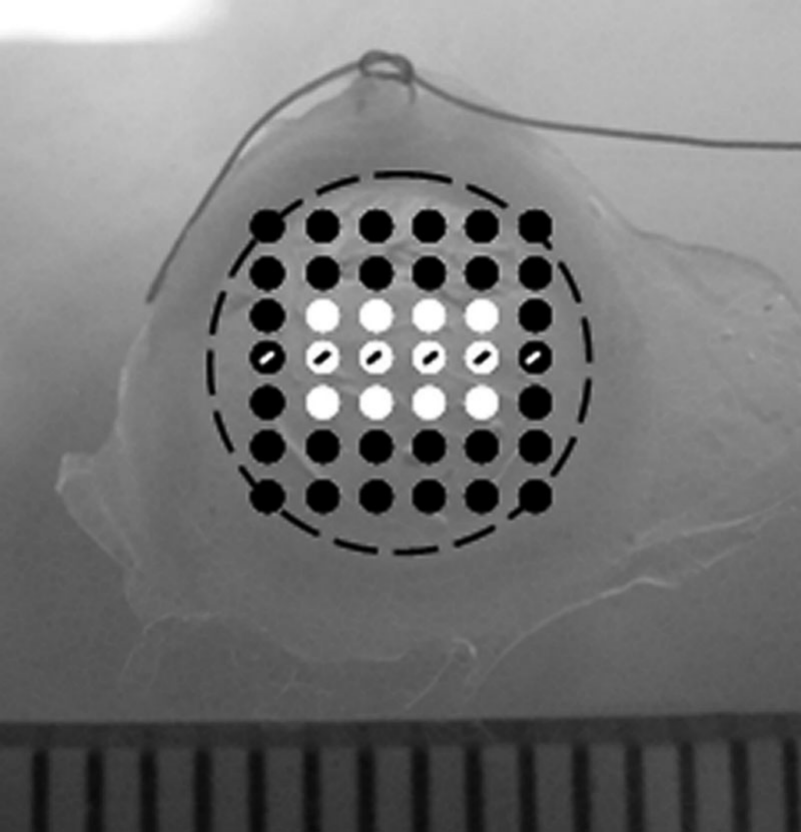
Small-angle X-ray scatter patterns (represented by circles) were collected at 1 mm intervals over each guinea pig cornea. Twelve measurements of fibril diameter, fibril separation distance, and D-periodicity from the central cornea were used (white circles) to produce weighted averages for each treatment group (Table 1). Measurements across the horizontal meridian of each cornea (circles containing a line) were averaged within treatment groups to show limbus-to-limbus changes in fibril separation distance and fibril diameter (Figure 3). The position of the limbus is shown as a dashed black line. A scleral suture at the 12 o’clock position ensured that in vivo corneal orientation was maintained during data collection.

Small-angle X-ray scattering data were obtained at 1 mm intervals (Figure 1) over 11 of the 16 corneas (Group A, n=3; Group B, n=2; Group C, n=3, Group D, n=3) on Station I22 at the Diamond Light Source, Oxford, UK. The patterns, each resulting from a 5 s exposure to a 1 Å wavelength X-ray beam with a cross-sectional area at the specimen of 0.25 mm×0.3 mm, were recorded on a RAPID2 detector positioned 6 m behind the camera. The system was calibrated against the meridional X-ray reflections arising from the axial 67 nm repeat of collagen in moist rat-tail tendon.

### X-ray data analysis

The X-ray scatter patterns were analyzed as described previously [19,21]. Unix-based software and statistics and graphics packages (Fit2D; European Synchrotron Radiation Facility, Grenoble, France; Excel; Microsoft Corp., Redmond, WA; and Statistica; Statsoft, Tulsa, OK) were used to produce measurements of the average Bragg fibril separation distance and fibril diameter from the respective positions of the intense equatorial reflection and the fainter subsidiary equatorial reflection. The axial D-period of corneal fibrillar collagen was calculated using the calibrated position of the third-order meridional reflection [19].

### Statistical analysis

Sample sizes for each group were: Group A, n=3; Group B, n=2; Group C, n=3; and Group D, n=3. The values presented in the results are means ± standard deviation (SD). Comparisons between treatment groups in terms of collagen parameters and corneal thickness measurements were statistically evaluated using ANOVA. In cases where the F-test p value was less than p<0.05, the null hypothesis was rejected and a pairwise comparison of means was performed using the least significant difference method. A difference between any pair of means of greater than or equal to the least significant difference at p<0.05 was considered to be statistically significant.

### Transmission electron microscopy

One intact cornea from each treatment was immersed in 2.5% glutaraldehyde/2% paraformaldehyde in 0.1 mol/l of Sörensen’s phosphate buffer at pH 7.4; after 1 h, the corneas were removed and blocks of approximately 1 mm×5 mm×full thickness were sampled from the peripheral and central corneal locations. These blocks were then returned to fixative for a further 3 h. After washing and storage in buffer, the blocks were postfixed in 1% osmium tetroxide, followed by 0.5% uranyl acetate, before dehydration in ethanol and embedding in Araldite CY212 epoxy resin (Agar Scientific, Stanstead, UK). Ultrathin sections (~90 nm) were collected on uncoated copper grids and contrasted with saturated uranyl acetate and lead citrate before examination in a Jeol 1010 transmission electron microscope (Tokyo, Japan) equipped with a Gatan Orius SC1000 digital camera (Abingdon, UK).

A series of images were acquired at magnifications of 3,000×, 4,000×, 5,000×, 6,000×, and 12,000× from outer, mid, and posterior stromal sites in the central and peripheral regions of the cornea.

## Results

### Physical characteristics and growth rate

Physical examination of the guinea pigs revealed changes in the ear skin of all irradiated animals (Groups C and D, data not shown) and the typical signs of low ascorbic acid intake (loss of weight and vitality) were seen in the ascorbic acid–deficient groups (B and D).

**Figure 2:**
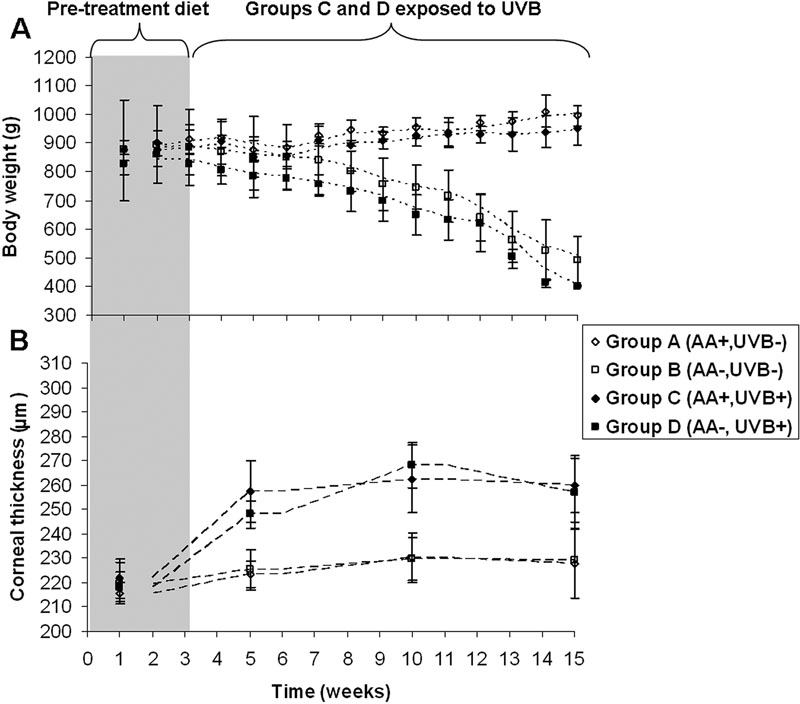
Changes in guinea pig body weight and corneal thickness over 15 weeks. **A**: Average (±standard deviation) body weight of guinea pigs fed a normal (AA+) or ascorbic acid deficient deficient (AA-) diet, with (UVB+) or without (-) daily UVB exposure. From week 9 onwards body weight was significantly lower in the ascorbic acid deficient animals (p<0.01).  **B**: Average (±standard deviation) corneal thickness measurements from the same guinea pigs revealed a significant increase in corneal thickness following UVB-exposure (p<0.01). Moving average trend lines show the pattern of animal growth and corneal thickness change during the course of the study.

Between weeks 1 and 7, there was no difference between the average bodyweight of animals fed a normal diet (Groups A and C) or an ascorbic acid–deficient diet (Groups B and D) (Figure 2A). However, after 8 (Group D) to 9 (Group B) weeks on an ascorbic acid–deficient diet, the animals in Groups B and D were found to have a significantly lower bodyweight than those maintained on a normal ascorbate diet (Groups A and C; p<0.05). Between weeks 8 and 15, the difference in bodyweight between normal (Groups A and C) and ascorbic acid–deficient animals (Groups B and D) continued to increase (p<0.01); animals fed a normal diet increased their bodyweight from 895 g to 950 g, while ascorbic acid–deficient animals experienced weight loss (from 730 g to 490 g). Daily exposure to UVB did not affect bodyweight.

### Corneal pachymetry and biomicroscopy

When measured at 5, 10, and 15 weeks, corneal thickness was significantly higher in the UVB-irradiated groups (Groups C and D) than in the nonirradiated groups (Groups A and B; p<0.001; Figure 2B). Throughout the study, corneal thickness did not differ significantly between the left and right eyes of individual animals, and in Groups A, C, and D corneal thickness did not vary significantly between each pair of animals exposed to the same treatment. However, a significant difference in corneal thickness was evident between the two ascorbate-deficient, nonirradiated animals in Group B (p<0.01).

Consistent with previous studies using the same housing environment [22], punctuate and linear superficial erosions were observed in the corneas of all animals. Each cornea examined showed positive fluorescein staining (arbitrary units 1 or 2), indicating mild to moderate uptake of the dye. No significant differences in fluorescein staining were detected between study groups.

### X-ray scattering

**Figure 3:**
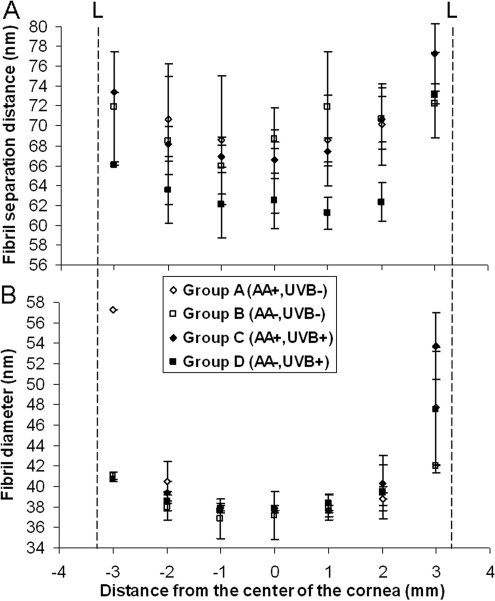
Changes in fibril separation distance and fibril diameter across the horizontal meridian of guinea pig corneas. **A**: Average (±standard deviation) fibril separation distance across the corneas of animals fed a normal (AA+) or ascorbic acid deficient deficient (AA-) diet, with (UVB+) or without (-) daily UVB exposure (Groups A: n=3; B: n=2; C: n=3; D: n=3). In the UVB-exposed corneas only,  fibril separation distance was significantly lower in the central 4 mm of the cornea than in the peripheral cornea (p<0.01). **B**: Average (±standard deviation) fibril diameter measurements across the same corneas showed an increase in fibril size with proximity to the limbus (L). p<0.05.

Within each treatment group, the diameter of collagen fibrils was lowest in the central 4 mm of the cornea, but increased with proximity to the limbus (Groups A and B; p<0.01 and Groups C and D; p<0.05; Figure 3). Although there was a trend for the fibril separation distance to be lower in the central 4 mm of the cornea than in the remaining peripheral cornea, this was only found to be significant in the UVB-exposed corneas (Group C and Group D; p<0.01).

By averaging 12 measurements of fibril separation distance, fibril diameter, and D-periodicity from the central region of each cornea (as shown in Figure 1), averages of each collagen parameter were calculated for all treatment groups (Table 1). On average, fibril separation distance was significantly higher in animals fed a normal level of ascorbic acid with (Group C) or without (Group A) exposure to UVB than in animals fed an ascorbic acid–deficient diet (Groups B and D; Table 1). Animals maintained on a normal ascorbic acid diet (Groups A and C) showed no difference in fibril separation distance as a result of UVB exposure. However, in ascorbic acid–deficient animals (Groups B and D), fibril separation distance was significantly lower in those exposed to UVB (Group D). The lowest fibril separation distance was seen when, as in Group D, the two treatments were combined (scorbutegenic diet and UVB exposure).

**Table 1:** The effect of Ultraviolet-B (UVB) exposure and normal (+)/deficient (-) dietary ascorbic acid (AA) on the average fibril separation distance, fibril diameter and D-period of stromal collagen in the guinea pig cornea.

**Specimen**	**FSD (nm)**	**Mean (nm)**	**Diameter (nm)**	**Mean (nm)**	**D-period (nm)**	**Mean (nm)**
**Group A (AA+,UVB-)**						
E*	69.1±1.9	68.1±4.5a	39.3±1.5	38.7±1.3e	64.2±0.5	64.2±0.5
F*	72.5±1.9		38.5±1.1		64.2±0.5	
G	62.7±2.1		38.5±1.4		64.2±0.5	
**Group B (AA-,UVB-)**						
H	66.7±2.7	65.9±2.5b	38.8±0.5	37.7±1.3 f	64.2±0.5	64.2±0.5
I	65.1±2.0		36.6±0.7		64.2±0.5	
**Group C (AA+,UVB+)**						
J*	68.7±1.3	67.5±2.6c	38.1±1.1	38.2±0.9g	64.2±0.5	64.2±0.5
K*	64.6±1.0		38.0±0.3		64.2±0.5	
L	69.2±2.3		38.5±1.0		64.2±0.5	
**Group D (AA-,UVB+)**						
M	59.6±1.4	62.5±2.7d	38.2±0.6	38.5±0.9h	64.2±0.5	64.2±0.5
N*	64.6±2.3		38.8±0.9		64.2±0.5	
O*	63.2±1.3		38.5±1.1		64.2±0.5	

Fibril separation distances (FSD), fibril diameters and D-periodicity are averages (±SD) of 12 measurements taken at equal intervals in the central region of each cornea. Means are the averages for each treatment group. Superscript letters a-h show where statistical differences exist between groups: a-d, b-d, c-d, e-f,=p<0.001; a-b, f-h=p<0.01; b-c=p<0.05. No significant differences existed between a-c, e-g, e-h, f-g and g-h. Asterisks are used to indicate left/right pairs of corneas within a treatment group.

In animals fed a normal level of ascorbic acid, exposure to UVB did not affect the average diameter of corneal collagen fibrils (Groups A and C). Furthermore, no change in fibril diameter was detected between animals in control Group C (normal ascorbate, no UVB) and those in Group D, which experienced both ascorbate deficiency and UVB exposure. Therefore, the relatively small diameter of collagen fibrils in Group B (low ascorbate, no UVB) compared to Groups A (normal ascorbate, no UVB) and D (low ascorbate, UVB exposed) appears to be something of an anomaly. This result may be attributed to the small sample size and large within-group variability in Group B (n=2), coupled with the difficulty associated with measuring average fibril diameter from the broad subsidiary maximum of the X-ray scatter pattern (estimated error of ±0.2 nm).

Collagen D-periodicity was unaffected by the level of ascorbic acid in the diet or by UVB exposure.

### Transmission electron microscopy

**Figure 4:**
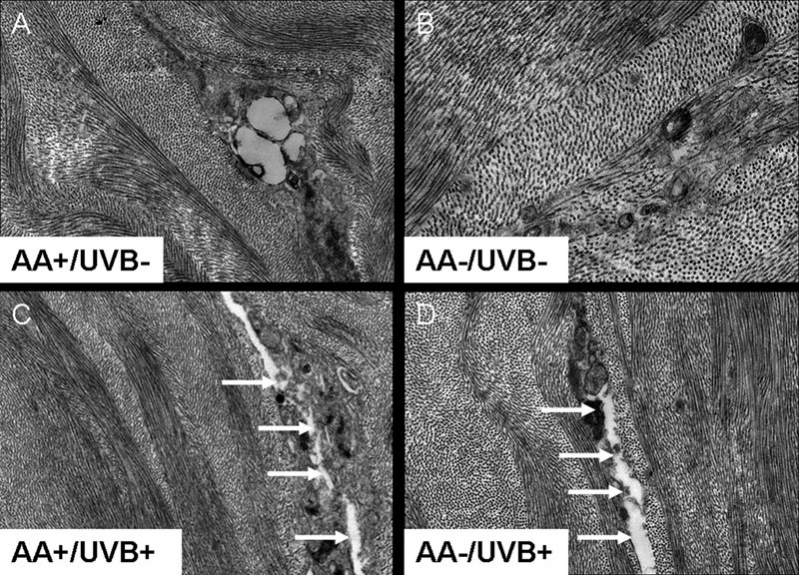
A selection of transmission electron micrographs from the corneal stroma of guinea pigs fed a normal (AA+) or ascorbic acid deficient (AA-) diet, with (UVB+) or without (UVB-) daily UV-B exposure. No clear differences in collagen fibril organization were evident between treatment groups and a disruption of cellular organelles was seen in all corneas (**A**-**D**). Regions devoid of regularly arranged collagen fibrils (arrows) were particularly evident in the UVB-treated corneas (**C** and **D**). Original magnification: 3000× (**A**, **C**, **D**) and 5000× (**B**).

Corneas from animals receiving normal dietary ascorbate and no UVB exposure (Group A) exhibited normal lamellar stromal structure with evenly spaced collagen fibrils (Figure 4A). However, this was also the case in nonirradiated ascorbate-deficient animals (Group B) (Figure 4B) and after UVB exposure, with (Figure 4C), or without (Figure 4D) normal ascorbate (Groups C and D). All corneas displayed some evidence of keratocyte disruption, including swelling of the endoplasmic reticulum and mitochondria and cell blebbing, allowing the release of membrane-bound vesicles and organelles into the extracellular matrix. However, collagen-free regions called “lakes” were particularly evident in the UVB-treated corneas (Figure 4C and D).

We cannot exclude the possibility that the freeze–thaw procedures used before tissues were fixed for transmission electron microscopy may also have given rise to some cell disruption. In addition, the fixative used for transmission electron microscopy may have masked the compaction of collagen fibrils observed using X-ray scattering. No clear-cut differences were detected in stromal structure between the central and peripheral locations.

## Discussion

Although it is well known that vitamin C is essential for collagen synthesis [13], and reports in the literature show that it is present in the cornea [6-8], there are only suggestions and no direct evidence that vitamin C offers protection to the corneal stroma against UV-induced oxidative damage. This study directly tests the individual and combined effects of vitamin C deficiency and UVB-exposure on corneal ultrastructure.

The guinea pig is an excellent model for investigating the effect of dietary vitamin C levels on the cornea and other connective tissues, since, like humans, guinea pigs’ lack of L-gulonolactone oxidase results in an inability to synthesize ascorbic acid de novo. Consistent with previous studies, we have shown that the prolonged consumption of an ascorbic acid–deficient diet results in weight loss in guinea pigs [20,23], and UVB exposure causes the cornea to increase in thickness [20,24]. The individual and combined effects of ascorbic acid deficiency and chronic UVB exposure on the corneal stromal ultrastructure were assessed using two complimentary techniques, namely transmission electron microscopy and synchrotron X-ray scattering. Synchrotron X-ray scattering offers some advantages over electron microscopy in that large amounts of data can be gathered in a relatively short period of time, and the tissue can be studied in a close to natural state without the need for lengthy tissue preparation and fixation. Unfortunately, the significant costs associated with the technique and the limited access to synchrotron radiation sources means that studying large numbers of corneas is not a practical proposition. Therefore, when considering the use of small sample numbers (as in this study), the amount and quality of the information obtained from each sample must be taken into account. For instance, our X-ray beam has a cross-section of 0.09 mm^2^ and averages every fibril throughout the corneal thickness (about 0.25 mm), thereby sampling a minimum tissue volume of 0.0225 mm^3^ or ~2×10^16^ nm^3^. It would therefore take *hundreds of millions* of electron micrographs to measure the same number of collagen fibrils as can be measured with a single X-ray scatter pattern.

Although the corneas of guinea pigs are smaller and thinner than those of humans, they share the same basic structure, with the stroma occupying approximately 70% of the tissue thickness. Analysis of X-ray scatter patterns collected across the nasal-temporal meridian of human [21] and guinea pig corneas have shown that the diameter of stromal collagen increases rapidly at the limbus. In the human cornea, this increase in fibril diameter is accompanied by an increase in fibril separation distance, a reduction in collagen organization [25], and a change in proteoglycan composition (a decrease in keratan sulfate and the appearance of chondroitin sulfate) [25]. It has been suggested that the higher packing density of collagen in the central region of the human cornea may provide additional strength in a region of reduced tissue thickness, and the presence of smaller fibrils in this region may enhance tissue transparency [21]. With this in mind, the absence of any discernible difference in stromal thickness between the central and peripheral regions in the nonirradiated guinea pig cornea [22] may explain the absence of any significant changes in fibril separation distance between the two regions.

X-ray scattering studies have shown that in the normal cornea, a positive linear relationship exists between fibril separation distance squared and tissue hydration (and hence thickness), indicating that additional water entering the cornea goes preferentially between the fibrils rather than within them [26]. However, when the cornea swells, water does not fill the interfibrillar space uniformly, and it is thought that a proportion must enter another compartment. As a regular arrangement of collagen fibrils is needed to cause constructive interference of X-rays, these compartments must presumably be devoid of collagen or occupied by irregularly separated fibrils that do not contribute significantly to the formation of a recordable interfibrillar reflection (such as measured here) [26]. We have shown that UVB-induced stromal thickening (observed here and reported elsewhere [9,24,27]) is not accompanied by a widespread expansion of the collagen fibrillar array, and in the case of ascorbate-deficient animals, the stromal thickening is associated with a compaction of collagen fibrils. This suggests that changes in corneal thickness and fibril separation distance after UVB treatment are more likely caused by an expansion of collagen-free stromal compartments (such as those identified here by electron microscopy). It is hypothesized that these collagen-free stromal compartments may be regions of the tissue that were previously occupied by keratocytes before UVB-induced apoptosis (a well described consequence of UVB exposure [28]). In the case of the ascorbate deficient animals in particular, we believe that these compartments may be hygroscopic in nature—drawing water in from the surrounding extracellular matrix and reducing the average separation distance between collagen fibrils. Additionally, the greater disparity in fibril separation distance between the central and peripheral regions of irradiated corneas compared to nonirradiated corneas indicates that UVB-induced stromal thickening and/or the mechanism by which the thickening occurs (via an expansion of the fibrillar array or an expansion of collagen-free compartments) may not be uniform across the cornea.

The compaction of corneal collagen fibrils in irradiated, ascorbate-deficient animals was not evident when the corneas were examined using electron microscopy. Using the same technique, Wu et al. [23] also reported the absence of any major differences in corneal morphology between UVB-irradiated animals fed a normal or low ascorbic acid diet. This suggests that the fibril compaction evidenced by X-ray scattering may not be a ubiquitous change throughout the entire guinea pig stroma, and thus may be less readily detected by microscopy than by the X-ray technique, which averages throughout the total stromal thickness. It is also possible that the differences in fibril compaction between treatment groups were lost during the processing of corneas for transmission electron microscopy, as osmium tetroxide postfixation, ethanol dehydration, and resin embedding have all been shown to significantly alter corneal collagen fibril separation distance [29].

It has been proposed that reactive oxygen species in the cornea, such as those generated following exposure to UVB irradiation (hydrogen peroxide, singlet oxygen, and free radicals), may cause direct cleavage of stromal glycosaminoglycans, thus altering their physiologic properties and making them more susceptible to degradation by tissue enzymes [30]. They may also induce collagen aggregation and crosslinking, as well as altering collagen-fibroblast interactions, collagen solubility, and mechanical strength [31,32]. Based on X-ray scattering measurements of average collagen fibril diameter and transmission electron microscopy images of collagen at specific tissue depths, there was no evidence to suggest that in this instance, UVB exposure resulted in collagen aggregation. Furthermore, our current study revealed an absence of any detectable changes between treatment groups in terms of collagen D-periodicity. This suggests that over a 12-week study period, neither the axial stagger nor the tilt of the collagen molecules are affected by a deficiency in ascorbic acid or by a daily 40 min exposure to 0.12 J/cm^2^ UVB. However, it must be remembered that collagen turnover in the unwounded cornea is notably slow (over 17 months in rabbits) [33], and so the effect of these treatments on the synthesis of new collagen (in which ascorbic acid plays a vital role) could not be ascertained from this study.

It is believed that a fine balance exists between the level of reactive oxygen species and antioxidants in the cornea [34,35], but some species (including humans) can recycle oxidized vitamin C and can function with a lower level of ascorbic acid from the diet to satisfy metabolic demands [36]. Based on this concept, it seems feasible that if the level of reactive oxygen species were to increase as a result of corneal injury (e.g., microerosions, likely caused by the sawdust in-house bedding) [21] or chronic UVB exposure and/or the level of antioxidants was reduced as a consequence of ascorbic acid deficiency, then a reactive oxygen species/antioxidant imbalance might occur. Such an imbalance could result in oxidative damage and changes in the corneal ultrastructure. Indeed, the structural changes caused by UVB radiation, as measured here using X-ray scattering, were greatest in animals fed an ascorbic acid–deficient diet, thereby supporting the belief that ascorbic acid plays a vital role in protecting the corneal stroma from the harmful effects of UVB.

Climatic droplet keratopathy does not usually occur before the age of 40 years (~3.3 guinea pig equivalent years), and typically affects those who have been exposed to a lifetime of chronic UVB and intense winds carrying particles of dust, sand, or ice [3]; in addition, studies of disease severity in Africa found the diet of climatic droplet keratopathy patients to be deficient in nutrients [37,38]. It is likely, therefore, that a longer timeframe for the generation of ascorbate deficiency than used here (15 weeks, which equates to ~3 human years) and/or a means of accelerating collagen turnover (such as by corneal wounding) is needed to assess the full effect of ascorbic acid deficiency and chronic UVB irradiation on the stromal ultrastructure. Investigations such as these may further our understanding of the development of corneal haze in climatic droplet keratopathy.
